# Genome-Wide Association Study to Map Genomic Regions Related to the Initiation Time of Four Growth Stage Traits in Soybean

**DOI:** 10.3389/fgene.2021.715529

**Published:** 2021-09-14

**Authors:** Wenliang Yan, Benjamin Karikari, Fangguo Chang, Fangzhou Zhao, Yinghu Zhang, Dongmei Li, Tuanjie Zhao, Haiyan Jiang

**Affiliations:** ^1^Key Laboratory of Biology and Genetics and Breeding for Soybean, Ministry of Agriculture, State Key Laboratory for Crop Genetics and Germplasm Enhancement, National Center for Soybean Improvement, Nanjing Agricultural University, Nanjing, China; ^2^College of Artificial Intelligence, Nanjing Agricultural University, Nanjing, China; ^3^Department of Crop Science, Faculty of Agriculture, Food and Consumer Sciences, University for Development Studies, Tamale, Ghana; ^4^Institute of Agricultural Sciences in Jiangsu Coastal Region, Yancheng, China

**Keywords:** soybean, growth stage traits, environmental and genetic factors, quantitative trait nucleotide, genetic improvement

## Abstract

The time to flowering (DF), pod beginning (DPB), seed formation (DSF), and maturity initiation (DMI) in soybean (*Glycine max* [L.] Merr) are important characteristics of growth stage traits (GSTs) in Chinese summer-sowing soybean, and are influenced by genetic as well as environmental factors. To better understand the molecular mechanism underlying the initiation times of GSTs, we investigated four GSTs of 309 diverse soybean accessions in six different environments and Best Linear Unbiased Prediction values. Furthermore, the genome-wide association study was conducted by a Fixed and random model Circulating Probability Unification method using over 60,000 single nucleotide polymorphism (SNP) markers to identify the significant quantitative trait nucleotide (QTN) regions with phenotypic data. As a result, 212 SNPs within 102 QTN regions were associated with four GSTs. Of which, eight stable regions were repeatedly detected in least three datasets for one GST. Interestingly, half of the QTN regions overlapped with previously reported quantitative trait loci or well-known soybean growth period genes. The hotspots associated with all GSTs were concentrated on chromosome 10. *E2* (*Glyma10g36600*), a gene with a known function in regulating flowering and maturity in soybean, is also found on this chromosome. Thus, this genomic region may account for the strong correlation among the four GSTs. All the significant SNPs in the remaining 7 QTN regions could cause the significant phenotypic variation with both the major and minor alleles. Two hundred and seventy-five genes in soybean and their homologs in *Arabidopsis* were screened within ± 500 kb of 7 peak SNPs in the corresponding QTN regions. Most of the genes are involved in flowering, response to auxin stimulus, or regulation of seed germination, among others. The findings reported here provide an insight for genetic improvement which will aid in breeding of soybean cultivars that can be adapted to the various summer sowing areas in China and beyond.

## Introduction

Soybean (*Glycine max* [L.] Merr.) is a versatile crop with numerous nutritional qualities and uses. It is the highest source of plant protein and second highest oil yielder among oilseed crops (Kole et al., 2015). As a photoperiod-sensitive and self-pollinated species, the growth stage traits (GSTs) play important roles in the adaptability and yield improvement of soybean (Li M. et al., 2019). Among the GSTs, flowering time and maturity are important agronomic traits related to soybean productivity (Wang et al., 2018). Furthermore, characteristics related to pods and seeds are also often regarded as yield-related traits, or important components of yield in soybean (Du et al., 2017; Li M. et al., 2019; Song et al., 2020), which emphasizes the importance of understanding the genetics underlying the initiation time of GSTs.

The GSTs are controlled by both genetic (G) and environmental (E) factors, which make breeding efforts utilizing traditional, conventional methods both ineffective and inefficient, leading to limited progress having been made on soybean adaptability, biomass, and economic yield (Cober and Morrison, 2010). In addition, the genetic mechanisms governing soybean flowering time to maturity are complex, regulated by polygenes with both major and minor effects (Zhang et al., 2015). So far, several loci/genes have been reported to regulate flowering in soybean, prominent among them include the *E-*series (E1–11) (Bernard, 1971; Buzzell and Voldeng, 1980; Mcblain and Bernard, 1987; Kang and Kim, 1996; Bonato and Vello, 1999; Molnar et al., 2003; Watanabe et al., 2009; Cober et al., 2010; Kong et al., 2014; Samanfar et al., 2017; Wang et al., 2019), *J* (Ray et al., 1995), *Dt1* (Liu et al., 2008), and *Dt2* (Zhang D. et al., 2019). It has been reported that these loci interact with photoperiod in modulating flowering and maturity. Under natural day length, the dominant alleles tend to delay flowering time and maturity, but the magnitude of effect of each gene can be different.

To date, SoyBase database^1^ currently lists 356 quantitative trait loci (QTLs) for first flower, 11 QTLs for pod beginning (Tasma et al., 2001), and 5 QTLs for seed formation (Mansur et al., 1993; Palomeque et al., 2010). Genetic studies on the initiation traits of the soybean pod and seed are relatively few. The pod-bearing period is the most vigorous growth stage of soybean and also the maximum phase when dry matter forms, partitions, and accumulates (Hu et al., 2019). Reasonable pod-beginning time and as many pods as possible will directly lead to a high yield, especially in vegetable soybean varieties (Li X. et al., 2019). Tasma et al. (2001) mapped a major effect QTL for days until pod formation on chromosome six in two different soybean populations, IX132 and IX136. The QTL region was found between the marker *Satt205* and *P029D_2*, which accounted for as much as 36.5% total phenotypic variance in population IX132, and 44.4% of total phenotypic variance in population IX136. Asakura et al. (2012) in addition reported 29,788 genes expressed in soybean at four pod development stages via DNA microarray analysis. However, the QTLs or genes related to manipulating the pod formation trait of soybean are rarely reported on.

The seed-formation characteristic of soybean is also worthy of attention. Seed yield is the product of a number of yield related traits, including seed number and seed size, factors that are largely determined at early seed developmental stages (Ruan et al., 2012; Zhang et al., 2021). Jones and Vodkin (2013) found more than one hundred genes that were highly expressed exclusively at young seed stages, whereas Karikari et al. (2020) identified three hub genes (*Glyma06g44510*, *Glyma08g06420*, and *Glyma19g28070*) that were expressed relatively more highly in large-seeded cultivars than small-seeded cultivars at seed formation stage.

With the advance of next-generation sequencing in recent years, the genome-wide association study (GWAS) can be more efficiently applied in germplasm collections or naturally occurring populations and provides higher resolution in terms of defining the genomic positions of QTLs/genes (Shirasawa et al., 2013). In addition, the first whole-genome sequence of the variety Williams 82, completed in 2010, provides a powerful resource for soybean functional genomic research (Schmutz et al., 2010). Moreover, multi-locus GWAS methods, e.g., Fixed and random model Circulating Probability Unification (FarmCPU) (Liu et al., 2016), are more powerful and robust in detecting loci associated with polygenic and/or multi-factorial complex traits than single-locus methods (Zhang Y. et al., 2019; Sertse et al., 2021), where single-locus models tend to only be able to detect large-effect QTNs (He et al., 2019b).

Favorable alleles could be successfully introgressed by using marker-assisted selection (MAS), if they are independent of the environmental and genetic background (Palomeque et al., 2010). This means that genetic studies of the same trait in multiple environments seems more scientifically viable to identify stable quantitative trait nucleotides (QTNs) for practical plant breeding. In this study, we evaluated 309 diverse soybean accessions across six different agricultural experimental environments to record four GSTs [i.e., days to flowering (DF), pod beginning (DPB), seed formation (DSF), and maturity initiation (DMI)], then conducted a FarmCPU GWAS with individual environment and best linear unbiased prediction (BLUP) values by utilizing 61,174 single nucleotide polymorphism (SNP) markers to identify QTN regions. Also, we mined candidate genes around the stable QTN regions for functional genomics studies in order to unravel the molecular mechanisms underlying the studied GSTs. Our overall objective was to provide a valuable insight into the genetic architecture of soybean GSTs and genetic resources for accelerating soybean genomic breeding, aimed at yield and yield stability as well as wider adaptability to multiple ecological zones within China and beyond.

## Materials and Methods

### Plant Materials and Experiment Design

A set of 309 breeding lines adapted to the Chinese Yangtze-Huai region, primarily utilized for grain and vegetable were used in this experiment. This population is designated as Yangtze-Huai Soybean Breeding Line Population (YHSBLP). Seeds for the panel were obtained from the National Center for Soybean Improvement, Nanjing Agricultural University, Nanjing, in Jiangsu Province, China.

All soybean materials were planted via a randomized complete block design with three replications in six different environments across 2 years (2018 and 2019) viz., Jiangpu (JP) Experimental Station (32°12′N and 118°37′E), and Yancheng (YC) City (33°21′N and 120°09′E) in Jiangsu province, and Dangtu (DT) Experimental Station (31°34′N and 118°29′E) in Anhui province. 2018 planting was done on June 22 in YC and July 3 JP and these were designated 18YC and 18JP, respectively. 2019 planting was done on June 21 in DT (19DT), June 24 in JP (19JP), June 19 in YC (19YC6), and July 4 (19YC7), according to the local soybean cropping calendar. Standard cultural and agronomic practices were followed similarly in each environment.

### Phenotypic Evaluation

Four traits related to GSTs viz., DF, DPB, DSF, and DMI were recorded following the Fehr’s classification method (Fehr et al., 1971). Briefly, DF was recorded at the R1 stage (the day when 50% of the plants on a plot have an open flower at one of the top four nodes with a fully expanded leaf); DPB represents days from emergence to a ¼ inch (0.5 cm) long pod at one of the four uppermost nodes on the main stem with a fully developed leaf at the R3 stage; DSF refers to days from emergence to a 1/8 inch (0.3 cm) long seed in one pod present at any of the four uppermost nodes on the main stem with a fully developed leaf at the R5 stage, and DMI were recorded at the R7 stage (days from emergence to 50% of the leaves turning yellow and one pod having reached full mature coloration).

### Statistical Analysis

Descriptive statistics such as mean, standard deviation (SD), maximum and minimum trait value, and coefficient of variation (CV%) were calculated using SAS version 9.3 (SAS Institute, 2010, Inc., Cary, NC, United States). Analysis of variance (ANOVA) was conducted to evaluate the effects of genotype (G), environment (E), and genotype-by-environment interaction (G × E) on the RGS traits in the joint environments using the PROC general linear model (GLM) in SAS software. The statistical model for the ANOVA was *y*_*ijk*_ = μ + *G*_*i*_ + *E*_*j*_ + *GE*_*ij*_ + *R*_*k*(*j*)_ + ε_*ijk*_, where *y*_*ijk*_ stands for the individual observation of *ijk*^*th*^ experimental unit; μ is the total mean, *G*_*i*_ is the effect of the *i*th genotype, *E*_*j*_ is the effect of the *j*th environment, *GE*_*ij*_ is the interaction effect between the *i*th genotype and the *j*th environment, *R*_*k(j)*_ is the effect of the *k*th block within the *j*th environment, and ε_*ijk*_ is a random error following N(0,$\sigma_{e}^{2}$). Broad-sense heritability (*h*^2^) was calculated as: $h^{2}=\sigma_{g}^{2}/\left(\sigma_{g}^{2}+\frac{\sigma_{g\,e}^{2}}{n}+\frac{\sigma_{e}^{2}}{n\,r}\right)$ for the combined environments, where $\sigma_{g}^{2}$is the genotypic variance, $\sigma_{g\,e}^{2}$is the G × E interaction variance, $\sigma_{e}^{2}$is the error variance, n is the number of environments, and r is the number of replications. Pearson correlation coefficients among the traits were estimated and visualized with *Corrplot* package in R (Taiyun and Viliam, 2017).

To minimize the effects of environmental variation, the best linear unbiased predictions (BLUPs) of individual lines for each trait were calculated using the R package *lme4* (Bates et al., 2014). Frequency distribution of phenotypic BLUPs was plotted by using OriginPro 2020 Statistical Software (Origin Corporation, Northampton, MA, United States).

### SNP Genotyping

The present study used restriction-site-associated DNA sequencing (RAD-seq) approach to sequence the genomic DNA of 309 lines of YHSBLP, and this sequencing was carried out by Beijing Genomics Institution, Shenzhen, China. First, the genomic DNA of all 309 soybean lines was extracted from young and healthy leaf samples with a modified CTAB method (Allen et al., 2006). *Taq I* enzyme was used to digest this genomic DNA for the construction of a genomic DNA library. The DNA fragments of 400–700 bp were selected and sequenced using an Illumina HiSeq 2,000 standard protocol for multiplexed shotgun genotyping, and 90-mer paired-end reads were generated (Andolfatto et al., 2011). All the sequence reads were aligned to the reference genome of Williams 82 (genome assembly 1 annotation version 1.1) (Schmutz et al., 2010), using the SOAP2 software (Li et al., 2009). Based on the Bayesian estimation of the site frequency, RealSFS was utilized for the SNP calling (Yi et al., 2010). The SNP data was screened at a rate of missing and heterozygous allele calls ≤ 30%, and then the missing genotypes were imputed using fastPHASE software (Scheet and Stephens, 2006). A total of 61,174 SNPs with minor allele frequencies (MAF) ≥ 5% were selected from 87,308 SNPs and were used for the GWAS analysis. The SNP dataset used in the current study is available in the Sequence Read Archive (SRA) at NCBI (SRA accession: PRJNA648781) repository, and on the website of the National Center for Soybean Improvement.^2^

### Population Structure and Linkage Disequilibrium Analyses

SNPs were first pruned by the indep-pairwise command option of pLINK v1.07 (Purcell et al., 2007), and the pruned SNPs were used to infer population structure using the Bayesian Markov Chain Monte Carlo (MCMC) model in STRUCTURE v2.3.4 software (Pritchard et al., 2000). The number of presumed populations (*K*) was set from 1 to 11 using a burn-in of 10,000, and a run length of 100,000 and, each *K*-value was obtained with seven independent runs. The statistic “Δ*K*,” which indicates the most likely number of subpopulations, was calculated by following the Evanno method (Evanno et al., 2005), and then the Q matrix under the highest Δ*K*-value was obtained. A pairwise distance matrix derived from the Nei’s genetic distance for all polymorphic SNPs was calculated to construct a Neighbor-joining (NJ) tree using TASSEL 5.0, and the Kinship was also analyzed in this software (Bradbury et al., 2007). Principal component analysis (PCA) was performed by using GCTA v1.92 software (Yang et al., 2011).

Linkage disequilibrium (LD), estimated as the *r*^2^ between SNPs, was calculated for each chromosome based on the LD tools option of RTM-GWAS V1.1 (He et al., 2017). LD was visualized using the mean *r*^2^ within bin sizes of 100 kb within the 5 Mb for each chromosome (Chr.) and then averaged across the whole genome. LD decay was calculated as the point at which the chromosomes reached 50% of their original LD value (Huang et al., 2010). Only *r*^2^ for SNPs with pairwise distances less than 5 Mb in every chromosome was used to draw the average LD decay figure by GraphPad Prism version 5.01 (GraphPad Software, San Diego California United States).^3^

### Genome-Wide Association and Haplotype Block Analyses

A total of 61,174 SNPs with MAF >5% were utilized for the association mapping with the 309 soybean lines. The FarmCPU model was used to detect significant marker-trait associations in GAPIT version 3.0 (Lipka et al., 2012). The Kinship matrix (K) and pseudo QTNs were used as covariates to minimize false positives as well as to increase statistical power. The significance threshold used in the present study was set at −log_10_ (1/m) where m = the number of markers, thus −log_1__0_(1/61,174) = 4.787 as the Bonferroni correction line. The phenotypic variation explained (PVE) was calculated by mixed liner model in TASSEL (Bradbury et al., 2007). Manhattan plots were generated using the R software package *qqman* (Turner, 2018). Haplotype block analysis was carried out for the significant and stable QTN regions in Haploview software version 4.2 with four gamete rule using default settings (Barrett, 2009). The GSTs of accessions in each significant SNP were compared with Duncan Range Multiple test via SPSS 25.0 (IBM Corp. Released 2017, Armonk, NY: IBM Corp) at significant level of 0.05 to determine the extent of variation among the accessions grouped.

### Candidate Gene Prediction Within Major QTNs

For each of the four growth stage traits, there will be seven phenotypic datasets, including the measured data from six different environments plus the BLUP values. We treated SNPs that were co-localized in at least three datasets and exceeded the significance threshold as QTNs. Based on the LD distance, we extended and selected the region of 500 kb upstream and downstream of the QTNs as 1.0 Mb QTN regions (Brodie et al., 2016; Karikari et al., 2020). According to the functional annotations available in the TAIR^4^ and SoyBase (see text footnote 1) databases as well as available literature, the possible candidate genes were predicted within major significant QTN regions.

## Results

### Natural Variation Available in YHSBLP Population for RGS Traits

**FIGURE 1:**
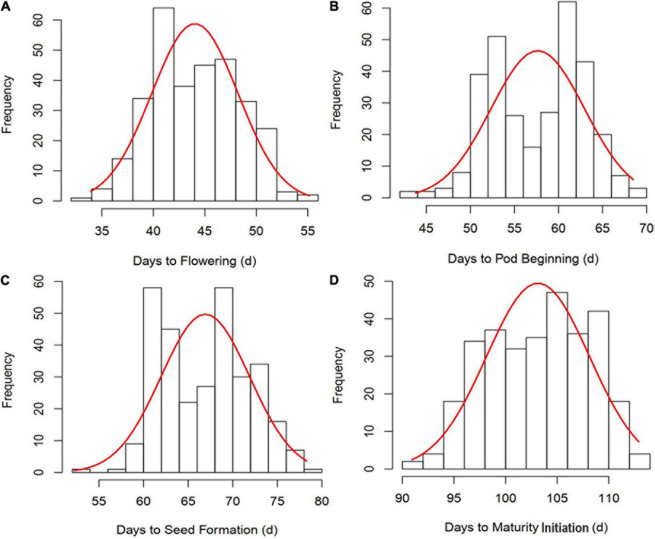
Frequency distribution and correlation analysis of four growth stage traits (GSTs) best linear unbiased prediction values (BLUPs). **(A–D)** represent the frequency distribution of time to flowering, pod beginning, seed formation and maturity initiation, respectively.

All the GSTs exhibited large phenotypic variation across the six environments. Descriptive statistics and *F*-values from ANOVA for the four GSTs showed the highest CV of 12.54% was observed for DF followed by DPB, DSF, and DMI (Table 1). The genotype by environment interaction (G × E) effects were significant (*P* < 0.05), revealing that phenotypic variation of GSTs are influenced by both genetics and environment. A significant positive correlation showed among the four GSTs with correlation coefficient (*r*) ≥ 0.86 with *P* < 0.01. Besides, for the individual environments’ analysis, an average highly significant correlation (*r* = 0.72, *P* < 0.001) was also shown among the GSTs’ measured data across all six environments (Supplementary Figure 1). These phenomena suggested that the four GSTs may share similar genetic bases. Figures 1A–D showed that the frequency distributions of the four GST-BLUP values followed normal distribution. The *h*^2^ for the four GSTs was high, in the range of 94.17–96.74%. These results suggest that selection based on only phenotypic observations may be misleading.

**TABLE 1:** Descriptive statistics for four growth stage traits across six different environments in the 309 soybean accessions.

Trait^*a*^	Mean	SD	Min	Max	CV(%)	*F*_*G*_ ^*b*^	*F*_*E*_ ^*b*^	*F*_*R(E)*_ ^*b*^	*F*_*GXE*_ ^*b*^	h^2^(%)^*c*^
DF	44	5.54	27	65	12.54	25.39***	5.63***	1.02	4.79***	96.74
DPB	58	6.72	40	79	11.62	24.2***	2.76**	0.4	4.6***	96.33
DSF	67	6.87	47	87	10.82	21.85***	1.73	0.97	4.53***	96.25
DMI	103	8.71	82	124	8.56	11.33***	1.12	0.28	3.93***	94.17

*^a^DF, days to flowering; DPB, days to pod beginning; DSF, days to seed formation; DMI, days to maturity initiation.*

^b^From ANOVA Table; F_G_, F_E_, F_R(E)_, and F_G × E_ represent the F-values for genotypic, environmental, block effects and genotype × environment interaction, respectively. **p < 0.01, ***p < 0.001.

*^c^Broad-sense heritability.*

### Population Structure and Linkage Disequilibrium Analysis

**FIGURE 2:**
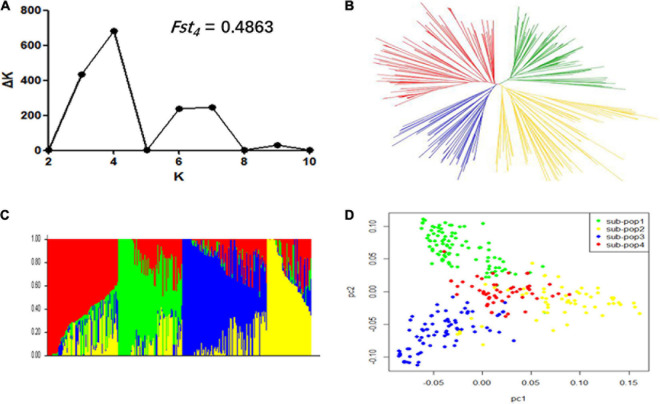
Population structure of 309 soybean accessions. **(A)** Calculation of the true *K* of the YHSBLP following procedure outlined by Evanno et al. (2005). **(B)** A neighbor-joining tree of the tested accessions that could be divided into four subpopulations. **(C)** Population structure derived from STRUCTURE software. Four colors (blue, yellow, green, and red) represent the four subpopulations (subpopulation 1, 2, 3, and 4, respectively). Each vertical column represents one individual and each colored segment in each column represents the percentage of the individual in the population. **(D)** Principal component analysis plot of the 309 accessions; two-dimensional scales were used to reveal population stratification.

In order to avoid false-positive associations from population stratification, we exploited three statistical methods viz., population structure, NJ based-tree, and PCA to estimate the relatedness among 309 accessions. The Δ*K* reached the highest value when *K* was at four (Figure 2A). This indicated that these 309 accessions could be divided into four subpopulations. The measurement of population differentiation, *F*_*ST*_, was estimated at 0.49 (*P* < 0.001) between the four subpopulations, suggest high level of genetic variation among our association panel. The results of NJ phylogenetic tree and PCA (Figures 2B,D) were consistent with the population stratification obtained from the STRUCTRUE software (Figure 2C).

**FIGURE 3:**
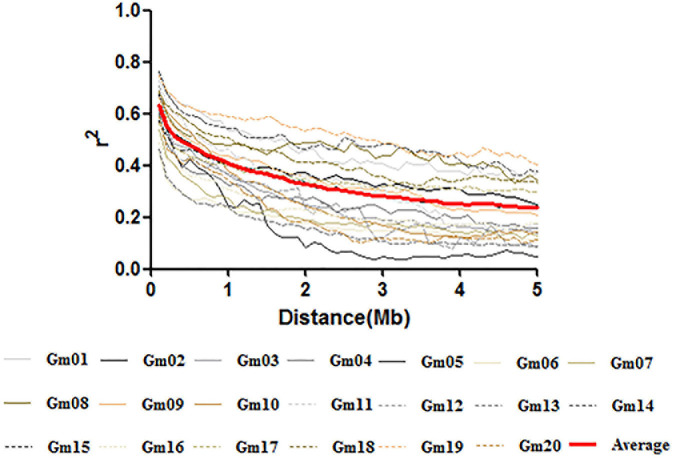
Linkage disequilibrium decay plots across soybean chromosomes and the average decay across the genome.

The LD decay rate varied considerably among the 20 chromosomes in soybean, and an average of 50% decay rate was observed between 800 kb and 3.2 Mb. However, except for Chr.08, Chr.14, Chr.17, and Chr.19, the *r*^2^ did not drop to half of its maximum value until 3.5 Mb; the average LD rate for SNPs reached 50% of this value at approximately 1.5 Mb across the whole genome (Figure 3).

### QTNs for Four GST Traits

**FIGURE 4:**
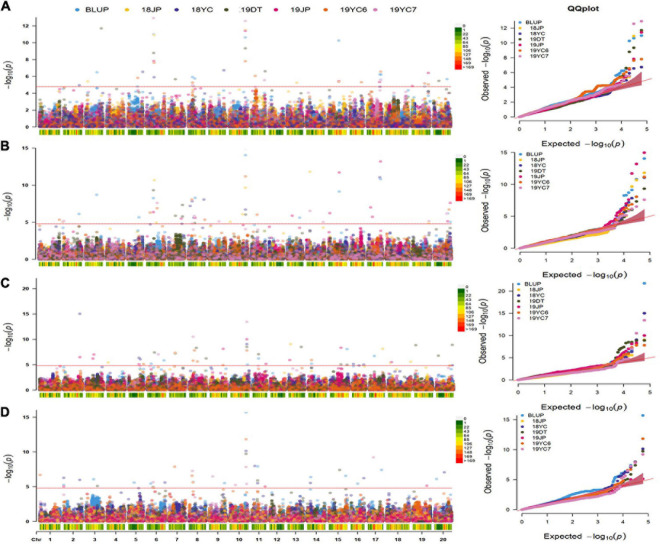
Manhattan and quantile–quantile plots for four GSTs in five different environments and BLUPs. **(A–D)** represent the Manhattan plots and Q-Q plots for the days to flowering, pod beginning, seed formation and maturity initiation, respectively. 18 and 19 represent the years of 2018 and 2019, DT : Dangtu; JP : Jiangpu; 2019YC6 and 2019YC7 means the planting date of June and July in Yancheng city, respectively. Red Line represents the significance threshold as determined by Bonferroni multiple comparisons corrections equivalent to –log_1__0_(*p*) = 4.787.

A multi-locus model, FarmCPU, was performed to identify significant SNPs associated with four GSTs. As a result, 50, 61, 55, and 47 SNPs were significantly associated [−*log*_10_ (*P*) ≥ 4.787] with DF, DPB, DSF, and DMI, respectively (Figures 4A–D and Supplementary Table 2). A total of 103 QTN regions were screened from the 213 SNPs by gathering them within a 1 Mb distance, and 8 of them were co-located in at least three datasets (Supplementary Table 3). QTN regions were named based on their physical location on the chromosome, for example, *qGm01-1* represented the first QTN region on chromosome 1, and *qGm02-3* represented the third QTN region on chromosome 2 (Table 2). Moreover, one QTN region on Chr.10 was repeatedly detected in all GSTs at least three datasets, which owned the average phenotypic variation explained (PVE) of 4.79, 5.57, 6.05, and 5.62% for DF, DPB, DSF, and DMI, respectively, was named *qGm-10-1*. Another region of *qGm15-1*, was associated with DF and DSF, which had the average PVE for the two GSTs of 1.54 and 2.49%, respectively.

**TABLE 2:** Eight QTN regions associated with four growth stage traits via FarmCPU method across different environments in summer soybean.

Region^*a*^	Chr	SNP^*b*^	Pos.(bp)^*c*^	No^*d*^	−log_1__0_(*P*)	PVE(%)^e^	Position(Mb)^*f*^	Effect	Trait_environment^*g*^
*qGm02-1*	2	Gm02_150932	150,932	2	6.27	1.97–4.91	0.15–0.16	1.56	DPB_6; DMI_1,2,3,5
*qGm06-1*	6	Gm06_16710123	16,710,123	1	5.65	1.28–3.12	16.71	2.11	DMI_1,4,7
*qGm06-2*	6	Gm06_19332290	19,332,290	1	12.93	1.44–5.77	19.33	−2.31	DF_7; DPB_1,4,5; DSF_4
*qGm06-3*	6	Gm06_21072696	21,072,696	2	10.91	2.74–4.13	20.99–21.07	−2.02	DF_2,3,6
*qGm10-1*	10	Gm10_44378814	44,378,814	3	21.76	2.39–7.66	44.35–44.55	−2.05	DF_1,2,4,5,6,7; DPB_1,2,3,4,5,6;
									DSF_1,3,4,5,6,7; DMI_1,3,4,6,7
*qGm11-1*	11	Gm11_15963231	15,963,231	3	7.92	2.04–3.70	15.95–15.96	−1.29	DPB_6; DSF_1,4; DMI_1,3,4
*qGm15-1*	15	Gm15_29990587	29,990,587	4	11.71	0.51–4.61	29.75–29.99	1.95	DF_1,4,5,6; DPB_1,5;
									DSF_1,4,5; DMI_4
*qGm17-1*	17	Gm17_37676700	37,676,700	2	7.41	2.40–3.26	37.68–37.79	1.06	DF_1,6,7; DPB_1

*^a^QTN regions which was detected at least in three environments.*

*^b^SNPs that were most significantly associated with the trait.*

*^c^QTN position (bp) on soybean genome assembly Wm82.a2.v1.*

*^d^The number of significant QTNs identified in the region.*

*^e^Phenotypic variation explained (PVE) calculated by mixed liner model.*

*^f^Interval range in each QTN region.*

*^g^The trait-environment combination of QTN. DF, days to flowering; DPB, days to pod beginning; DSF, days to seed formation; DMI, days to maturity initiation; 1–7 represent the environment code of BLUP, 18JP, 18YC, 19DT, 19JP, 19YC6, and 19YC7, respectively.*

Furthermore, the regions of *qGm06-3* and *qGm17-1* were mostly detected to associate with the DF trait within the remaining 6 QTN regions, which could contribute an average PVE of DF as 3.50 and 2.85%, respectively. Two QTN regions, *qGm02-1* and *qGm06-1*, were mostly associated with the DMI trait, and had average positive genetic effect as 1.15 and 1.86. Unprecedentedly, the *qGm06-2* region was identified to associate mostly with the DPB trait, and caused an average PVE of 4.05%, with the average negative genetic effect of −2.01. The final QTN region was *qGm11-1*, which associated with DMI in three datasets. It contributed an average negative genetic effect of −0.83 for this trait (Table 2 and Supplementary Table 3).

### Candidate Genes Within GSTs-Related QTN Regions

The most prominent SNP—i.e., *Gm10_44378814* in the *qGm10-1* region with the highest −log_1__0_(*P*) values (21.76) and associated with all four GSTs on Chr.10—overlapped with *E2* (*Glyma10g36600*), a well-known gene regulating flowering and maturation duration in soybean, and encodes a homolog of *GIGANTEA (GI)* gene (Watanabe et al., 2011). This gene is located 338 kb downstream from *Gm10_44378814* (Supplementary Table 4). A total of 275 Arabidopsis homologs genes within the remaining 7 QTN regions (except for the *qGm10-1* region) were screened (Supplementary Figure 2) and listed in Supplementary Table 5.

For the DF trait, the *qGm06-3*, *qGm15-1*, and *qGm17-1* regions harbor 47, 33, and 87 candidate genes, with expression data available for 22, 16, and 55 genes in the SoyBase transcriptome database (see text footnote 1). Among these genes, 6, 3, and 17 genes expressed more highly (by more than 50-fold) in flower tissues with the highest expression of fatty acyl-ACP thioesterases B protein (*Glyma06g23560*; 1,587-fold), followed by CTC-interacting domain 7 (*Glyma17g33510*; 287-fold), and Transcription factor TFIIE proteins (*Glyma06g23400*; 192-fold). For the DPB trait, *qGm6-2* region obtained 65 model genes, of which 28 had expression data on SoyBase (see text footnote 1). Six genes (*Glyma06g22410, Glyma06g22220, Glyma06g22840, Glyma06g22260, Glyma06g22660*, and *Glyma06g22800*) expressed higher in one-cm pod tissues with highest expression of COBRA-like extracellular glycosyl-phosphatidyl inositol-anchored protein family (*Glyma06g22410*; 216-fold), followed by a protein of unknown function (*Glyma06g22220*; 87-fold) and WD-40 repeat family proteins (*Glyma06g22840*; 66-fold). One DSF related QTN region of *qGm15-1* harbors 14 genes with available expression data from 33 model genes. Only 2 genes expressed more highly in seed tissues 21 days after flowering (according to the Table 1), with the highest expression being of NAD(P)-binding Rossmann-fold superfamily protein (*Glyma15g27630*; 126-fold), followed by a protein of unknown function (*Glyma15g27790*; 51-fold). Two DMI related QTN regions was *qGm02-1* and *qGm06-1*. 63 and 93 candidate genes were screened from them, respectively. 9 and 8 genes expressed higher in seed tissues 42 days after flowering. Two kinds of ribosomal protein (*Glyma06g20540*; 1,181-fold and *Glyma02g00540*; 335-fold) were the two most highly expressed proteins, followed by an organic cation/carnitine transporter 3 protein (*Glyma06g20500*; 220-fold).

## Discussion

### Four Soybean Growth Stage Traits Were Significantly Affected by Genetic-Environment Interaction

As a model crop for photoperiodic response research (Garner and Allard, 1920), the phenotypic variation in the GSTs of soybean was considerably influenced by environmental conditions. The four GSTs (DT, DPB, DSF, and DMI) of 309 summer soybean genetic resources in this study varied widely (Table 1) across the six different environments and BLUPs. The G × E played a significant role, suggesting that in addition to genetic factors, environmental factors such as photoperiod and temperature have direct influence on the four GSTs. Similar observations have been reported by Li M. et al. (2019). While the genetic analysis of G × E for target traits in crop breeding has become the emerging research direction, the genetic background of important GSTs in soybean need to be paid more attention for increasing the adaptivity of the crop to diverse environments.

### Selection Basis of Linkage Disequilibrium Decay Distance and GWAS Method

LD is one of the important factors affecting mapping resolution of GWAS analysis (Zhang et al., 2015). In the present study, a considerable number of SNP markers (>61,000) were used for the detection of marker-trait associated QTNs linked with various GSTs in the YHSBLP mapping panel. We observed a diverse range of LD among various soybean chromosomes with an average LD decay distance of 1.5 Mb (Figure 3), which is consistent with previous studies (Vuong et al., 2015; Zhang et al., 2015; Copley et al., 2018). Our study also confirmed that soybean possesses long LD decay distance, and suggested that marker-trait association identified in soybean can be directly used in MAS rather than map-based cloning. The high LD may be due to cleistogamous characteristics of soybean which have a strong influence on genomic homogeneity and reduced genomic variation, and this characteristic might become more sensitive during domestication practices, resulting in low genetic diversity and high LD (Lam et al., 2010). In addition, LD in a population is affected by historical recombination, mutations, allele frequency, genetic drift, founder effects, artificial selection during domestication, or breeding (Alqudah et al., 2020). The high LD may lead to numerous unrelated genes being screened out, therefore, some studies have proposed to use a cutoff of 500 kb, since enhancers and repressors may be as distant as 500 kb from their genes (Brodie et al., 2016; Karikari et al., 2020). By keeping this in view, we defined the range of candidate genes and QTNs as within a 500 kb genomic region of significant markers in both directions, downstream and upstream, in this study.

Two association study methodologies have been accomplished in the past 10 years due to the rapid development of genome sequencing technologies and phenotypic capacities. These are the classical single-locus GWAS methods based on the General Linear Model (GLM) and Mixed Linear Model (MLM), and recently developed multi-locus GWAS methods such as FarmCPU (Chen et al., 2021). The multi-locus methods consider the information of all loci simultaneously, and consequently do not require false discovery rate correction, leading to higher QTN detection power (Zhang Y. et al., 2019). In our study, quantile–quantile plots of the FarmCPU method indicated that the GWAS model is a reasonable one to deal with the association analysis of soybean GSTs. Xu et al. (2018), He et al. (2019a), and Chen et al. (2021) had evaluated the ability of FarmCPU with other GWAS methods via the association study for Maize starch pasting properties, Flax pasmo resistance, and *Medicago truncatula* seed size and seed composition: all the studies showed a powerful QTN identified efficiency of FarmCPU.

### Genetic Bases of Four GSTs in YHSBLP Population

Fifty percent of the QTN regions in this study overlapped with the previously reported QTLs or genes with known functions related to GSTs of soybean (Supplementary Table 4). *E2* is a homologous gene *GmGIA* of *Arabidopsis thaliana GIGANTEA* (*GI*). The flowering time of *E2* and its near-isogenic lines (e2) was similar at high latitude 43°N and middle latitude 36°N, indicating that the regulation of *E2* gene on flowering time of soybean might not only depend on photoperiod, so that it had a stronger geographical adaptability (Watanabe et al., 2011). Therefore, the *E2* gene has broad application prospects in breeding practice. In this study, the same location on Chr.10 near the *E2* gene was identified among the four GSTs in both BLUPs and measured data, which may be the basis for the strong genetic correlation among GSTs. Considering the role of the *E2* gene in pod and seed is rarely reported, the results of this study have a guiding significance for the further functional study of *E2* gene.

The allele variation of the most significant SNPs in each QTN region could cause significant changes in the corresponding phenotype, indicating that there may be candidate genes affecting soybean GSTs in the remaining 7 QTN regions (Supplementary Figure 3). On Chr. 06, two QTN regions *qGm06-1* and *qGm06-2* were associated with DMI and DPB, respectively. Zhang et al. (2015) identified two SNPs, *Gm06_16723946* and *Gm06_16167243*, that are associated with flowering time and days from flowering to maturity, respectively, whose location was close to the most significant SNP—*Gm06_16710123* of *qGm06-1—*in this study. A similar result was also found in the genetic study on the reproductive to vegetative growth period ratio in soybean by Wang et al. (2015). Fang et al. (2017) found a region from 19,178,035 to 20,299,454 bp on Chr.06 was associated with full bloom date traits, which was overlapped with the region of *qGm06-2* detected in this study. Another QTN region, *qGm17-1* was located on Chr.17 associated with DF, the most significant SNP *Gm17_37676700* in this region was only 100 kb (*Gm17_37574384*) away from the genetic location results of soybean flowering time under various photo-thermal conditions reported by Mao et al. (2017).

Some candidate genes around the QTN regions are also noteworthy: *ARF4* (auxin response factor 4) and *GCR1* (G-protein-coupled receptor 1) are well-known genes modulating flowering, whose mutants affect the response to auxin and shorten time to flowering (Colucci et al., 2002; Pashkovskiy et al., 2016). We identified two orthologs of *ARF4* and *GCR1*, *Glyma06g23830 (At5g60450)* on Chr.06, and *Glyma17g33480 (At1g48270)* on Chr.17, which are located at the DF-related QTN regions of *qGm06-3* and *qGm17-1*, respectively. In addition, the *AGL8* (*At5g60910.1*) ortholog *Glyma06g22650* was detected on Chr.06 around the peak SNP Gm06_19332290 in the *qGm06-2* region, which was associated with DPB. *AGL8* is a MADS box gene and involved in the early step of specifying floral meristem identity as well as the later step of determining the fate of floral organ primordia (Mandel and Yanofsky, 1995; Balanzà et al., 2019), hence *Glyma06g22650* may be involved in regulating GSTs. A plant-specific HD2 histone deacetylase gene, *HD2B* (*At5g22650*), was identified around the peak SNP Gm11_15963231 on the *qGm11-1* region associated with DSF. *HD2B* was regarded as a genetic factor associated with seed dormancy and plant growth, development, and response to abiotic stresses (Yano et al., 2013; Han et al., 2016). Another *Arabidopsis* trehalose biosynthesis gene, *TPS1*(*At1g78580*), has an expression analysis and a spatial and temporal activity of promoter which suggests that this gene is active within the seed-filling stage of development., *TPS1* was related to *Glyma15g27480* in the *qGm15-1* region, which may have an influence on the seed formation time of soybean (Dijken et al., 2004; Vuong et al., 2015).

In the two remaining QTN regions associated with DMI, the *LFR* (*At3g22990*) gene coded an ARM repeat superfamily protein and was identified in the *qGm02-1* region. Its ortholog in rice, *OsLFR*, resulted in homozygous lethality in the early seed stage through endosperm and embryo defects via its depletion (Qi et al., 2020). Another gene, *ARR6 (At3g47620)* was reported as a response regulator of cytokinins, which could regulate the response to cytokinin stimulus and thus delay leaf senescence. This gene was found as an ortholog of *Glyma06g20660* in the *qGm06-1* region (Hallmark and Rashotte, 2020).

The markers detected in this present study could be validated and used for MAS geared at improving GSTs in soybean, and provide foundation for future projects, including the designing of Kompetitive Allele Specific PCR (KASP) markers for practical breeding programs. The candidate genes predicted provide valuable information for functional validation to elucidate the molecular mechanism underlying the studied traits.

## Summary

In this study, we evaluated 309 diverse soybean breeding lines across five different environments for four GSTs (DF, DPB, DSF, and DMI), and applied a FarmCPU GWAS with high-density SNPs (>61 k). Fifty-eight SNPs within 8 QTN regions were detected to associate with the four GSTs. In addition, one QTN region on Chr.10 was detected repeatedly among all four GSTs and overlapped with the well-known *E2* gene. Seven remaining QTN regions contained the peak SNPs of *Gm02_150932*, *Gm06_16710123*, *Gm06_19332290*, *Gm06_21072696*, *Gm11_15963231*, *Gm15_29990587*, and *Gm17_37676700* on Chr.02, Chr.06, Chr.11, Chr.15, and Chr.17 were identified uniquely or commonly associated with the four GSTs and caused significant phenotypic variation with both major and minor alleles. Two hundred and seventy-five *Arabidopsis* genes involved in defense response, flowering, embryo development and so on with their homologs in soybean were screened, and some predicted as candidate genes (*Glyma06g23830, Glyma17g33480, Glyma06g22650, Glyma15g27480*, and *Glyma06g20660*) for DF, DPB, DSF, and DMI of summer soybean. However, the predicted candidate genes need further screening and functional validation to ascertain their actual roles in modulating the GSTs in soybean.

## Data Availability Statement

The datasets presented in this study can be found in online repositories. The names of the repository/repositories and accession number(s) can be found in the article/Supplementary Material.

## Author Contributions

TZ and HJ conceived and designed the experiments. FC, FZ, YZ, and WY performed the experiments. DL and WY analyzed the data. WY drafted the manuscript. TZ and BK revised the manuscript. All authors contributed to the article and approved the submitted version.

## Conflict of Interest

The authors declare that the research was conducted in the absence of any commercial or financial relationships that could be construed as a potential conflict of interest.

## Publisher’s Note

All claims expressed in this article are solely those of the authors and do not necessarily represent those of their affiliated organizations, or those of the publisher, the editors and the reviewers. Any product that may be evaluated in this article, or claim that may be made by its manufacturer, is not guaranteed or endorsed by the publisher.

## Footnotes

1
https://www.soybase.org/

2
http://ncsi.njau.edu.cn/zygx.htm

3
www.graphpad.com

4
https://www.arabidopsis.org/

## Supplementary Material

Supplementary Figure 1 Pearson correlation among the four growth stage traits of the BLUPs **(A)** and measured data from six different environments **(B)**. ****p* < 0.001. DF- days to flowering; DPB-days to pod beginning; DSF- days to seed formation; DMI- days to maturity initiation. 1–7 represent the environment code of BLUP, 18JP, 18YC, 19DT, 19JP, 19YC6, and 19YC7, respectively.

Supplementary Figure 2 Manhattan plot and linkage disequilibrium block of 7 peak SNPs in different environments. Linkage disequilibrium blocks associated with flowering, pod beginning, seed formation and maturity initiation time near *Gm02_150932*, *Gm06_16710123*, *Gm06_19332290*, *Gm06_21072696, Gm11_15963231*, *Gm15_29990587*, and *Gm17_37676700*. Significance threshold is denoted by the red line which was set as –log_1__0_(*p*) = 4.787. The up panel was the Manhattan plots of negative log_10_ transformed *p-*values vs. QTNs. The down panel was haplotype block based on pairwise linkage disequilibrium *r*^2^-values. DF- days to flowering; DPB-days to pod beginning; DSF- days to seed formation; DMI- days to maturity initiation. 1–7 represent the environment code of BLUP, 18JP, 18YC, 19DT, 19JP, 19YC6, and 19YC7, respectively.

Supplementary Figure 3 Phenotypic variation between cultivars carrying different alleles of the QTNs significantly associated with four growth stage traits in various environments. The box plot shows the significant difference of days to flowering, pod beginning, seed formation and maturity initiation of the cultivars carrying two alleles of the QTNs. The significant QTNs were *Gm02_150932*, *Gm06_16710123*, *Gm06_19332290*, *Gm06_21072696*, *Gm11_15963231*, *Gm15_29990587*, and *Gm17_37676700*. The major allele of significant loci was marked by gray, and the minor allele was marked by pink. Significant differences tested by the *t*-test are also given (****p* < 0.001, ** *p* < 0.01, * *p* < 0.05). DF- days to flowering; DPB-days to pod beginning; DSF- days to seed formation; DMI- days to maturity initiation. 18 and 19 represent the years of 2018 and 2019, DT : Dangtu; JP : Jiangpu; 2019YC6 and 2019YC7 means the planting date of June and July in Yancheng city, respectively.

Supplementary Table 1 Phenotypic data of four growth stage traits in 309 summer soybeans across different datasets.

Supplementary Table 2 SNP associated with four GSTs in summer soybeans.

Supplementary Table 2.1 SNP associated with days to flowering of summer soybeans.

Supplementary Table 2.2 SNP associated with days to pod beginning in summer soybeans.

Supplementary Table 2.3 SNP associated with days to seed formation in summer soybeans.

Supplementary Table 2.4 SNP associated with days to maturity initiation in summer soybeans.

Supplementary Table 3 Details of eight QTN regions associated with four growth stage traits via FarmCPU method in summer soybeans.

Supplementary Table 4 The overlapped QTLs or genes between present and previous studies within ± 500 kp upstream or downstream from the peak SNPs.

Supplementary Table 5 The detailed functional annotation information of 275 candidate genes in or near the QTN regions.
