# Supplementary material for: Genome-Wide Association Study to Map Genomic Regions Related to the Initiation Time of Four Growth Stage Traits in Soybean
Source: Front Genet. 2021 Sep 14;12:715529. doi: 10.3389/fgene.2021.715529 (PMC8476948; doi:10.3389/fgene.2021.715529)
Supplement: Supplementary Figure 1 — Pearson correlation among the four growth stage traits of the BLUPs (A) and measured data from six different environments (B). ***p < 0.001. DF- days to flowering; DPB-days to pod beginning; DSF- days to seed formation; DMI- days to maturity initiation. 1–7 represent the environment code of BLUP, 18JP, 18YC, 19DT, 19JP, 19YC6, and 19YC7, respectively. [file Data_Sheet_1.docx]

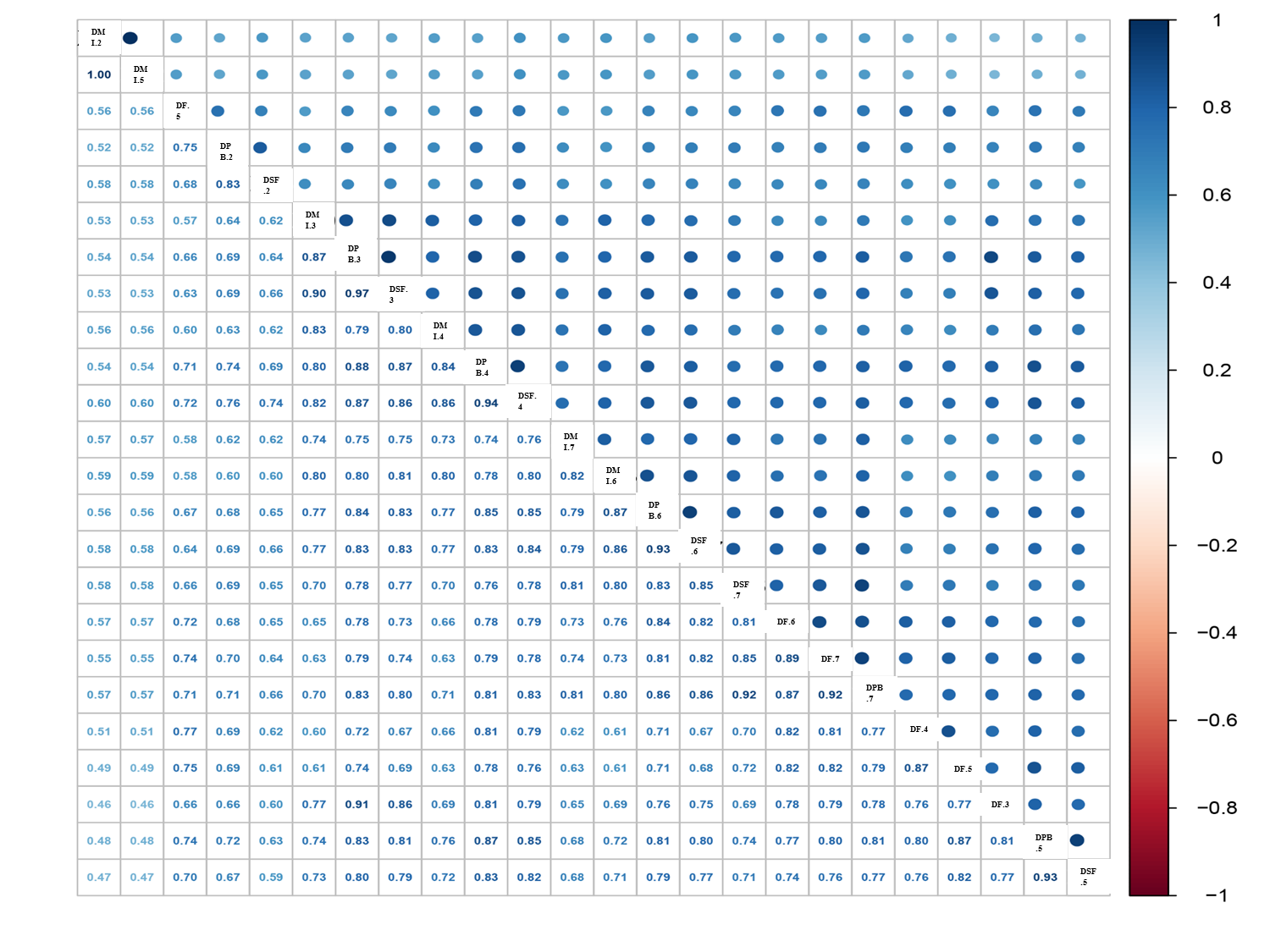

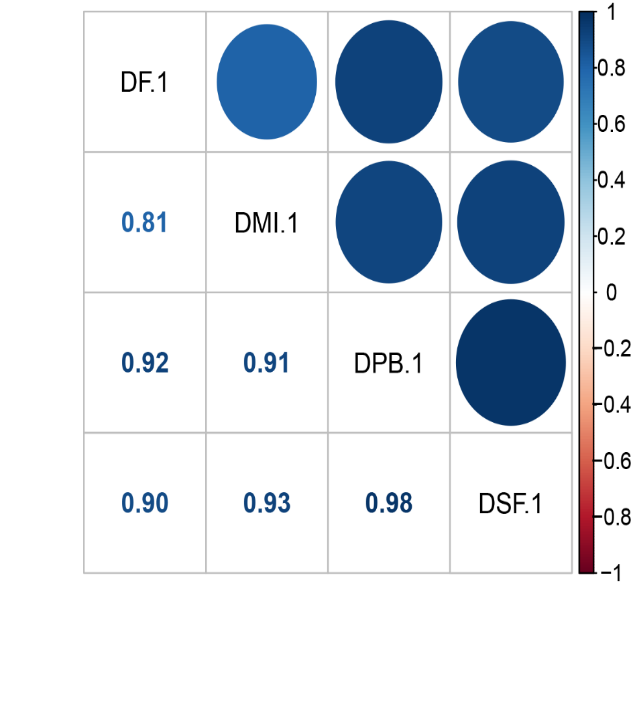


**A**

**B**

**Supplementary Figure 1.** Pearson correlation among the four growth stage traits of the BLUPs (A)and measured data from six different environments (B). ***p < 0.001. DF- days to flowering; DPB-days to pod beginning; DSF- days to seed formation; DMI- days to maturity initiation. 1-7 represent the environment code of BLUP, 18JP, 18YC, 19DT, 19JP, 19YC6 and 19YC7, respectively.
