# Supplementary material for: Genome-Wide Association Study to Map Genomic Regions Related to the Initiation Time of Four Growth Stage Traits in Soybean
Source: Front Genet. 2021 Sep 14;12:715529. doi: 10.3389/fgene.2021.715529 (PMC8476948; doi:10.3389/fgene.2021.715529)
Supplement: Supplementary Figure 2 — Manhattan plot and linkage disequilibrium block of 7 peak SNPs in different environments. Linkage disequilibrium blocks associated with flowering, pod beginning, seed formation and maturity initiation time near Gm02_150932, Gm06_16710123, Gm06_19332290, Gm06_21072696, Gm11_15963231, Gm15_29990587, and Gm17_37676700. Significance threshold is denoted by the red line which was set as –log10(p) = 4.787. The up panel was the Manhattan plots of negative log10 transformed p-values vs. QTNs. The down panel was haplotype block based on pairwise linkage disequilibrium r2-values. DF- days to flowering; DPB-days to pod beginning; DSF- days to seed formation; DMI- days to maturity initiation. 1–7 represent the environment code of BLUP, 18JP, 18YC, 19DT, 19JP, 19YC6, and 19YC7, respectively. [file Data_Sheet_2.docx]

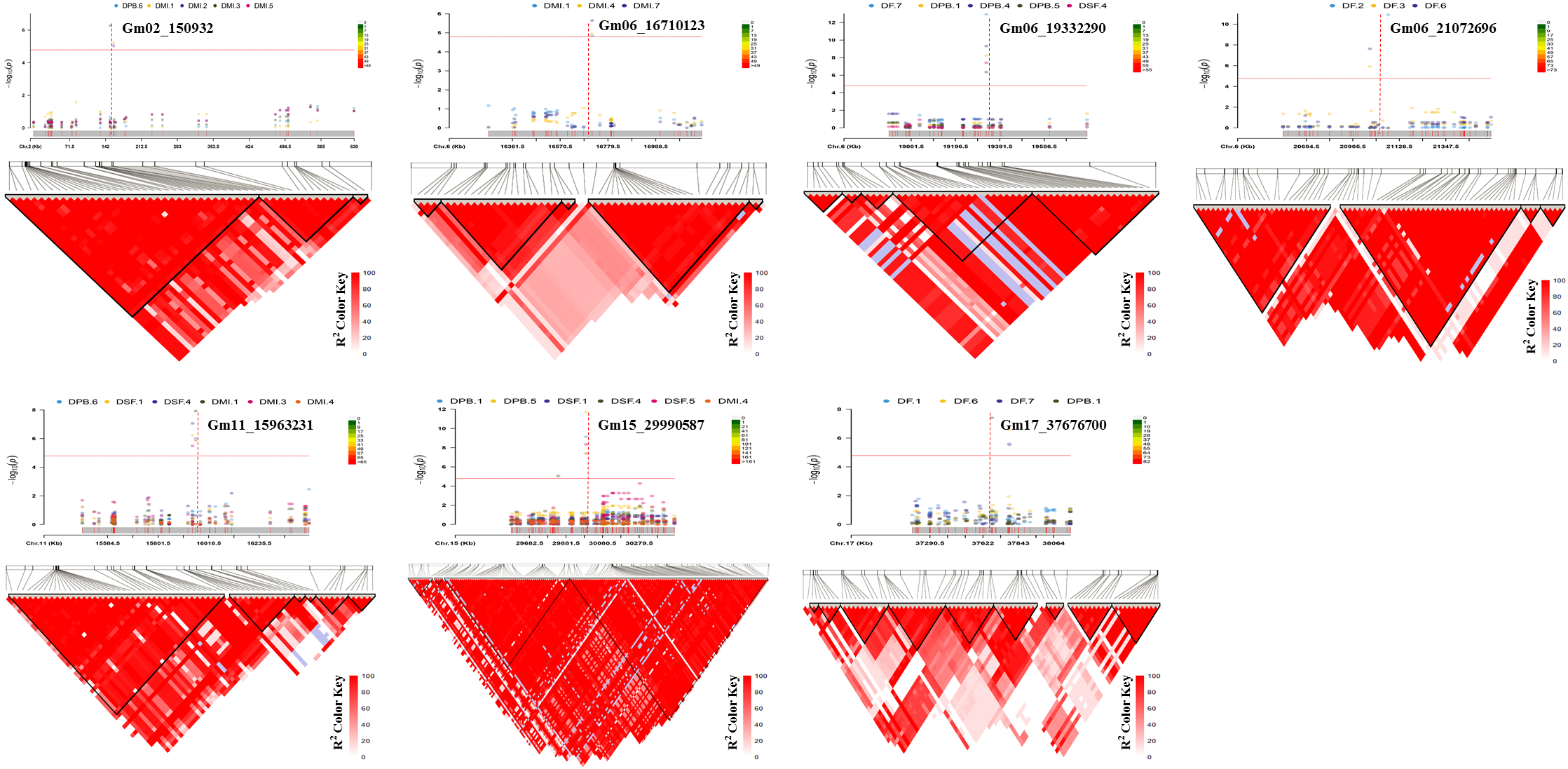


**Supplementary Figure 2. Manhattan plot and linkage disequilibrium block of 7 peak SNPs in different environments.** Linkage disequilibrium blocks associated with flowering, pod beginning, seed formation and maturity initiation time near *Gm02_150932*, *Gm06_16710123*, *Gm06_19332290,* *Gm06_21072696*, *Gm11_15963231*, *Gm15_29990587* and *Gm17_37676700*. Significance threshold is denoted by the red line which was set as –log_10_(*p*) = 4.787. The up panel was the Manhattan plots of negative log_10_ transformed *p-*values vs. QTNs. The down panel was haplotype block based on pairwise linkage disequilibrium r^2^ values. DF- days to flowering; DPB-days to pod beginning; DSF- days to seed formation; DMI- days to maturity initiation. 1-7 represent the dataset code of BLUP, 18JP, 18YC, 19DT, 19JP, 19YC6 and 19YC7, respectively.
