# Supplementary material for: Genome-Wide Association Study to Map Genomic Regions Related to the Initiation Time of Four Growth Stage Traits in Soybean
Source: Front Genet. 2021 Sep 14;12:715529. doi: 10.3389/fgene.2021.715529 (PMC8476948; doi:10.3389/fgene.2021.715529)
Supplement: Supplementary Figure 3 — Phenotypic variation between cultivars carrying different alleles of the QTNs significantly associated with four growth stage traits in various environments. The box plot shows the significant difference of days to flowering, pod beginning, seed formation and maturity initiation of the cultivars carrying two alleles of the QTNs. The significant QTNs were Gm02_150932, Gm06_16710123, Gm06_19332290, Gm06_21072696, Gm11_15963231, Gm15_29990587, and Gm17_37676700. The major allele of significant loci was marked by gray, and the minor allele was marked by pink. Significant differences tested by the t-test are also given (***p < 0.001, ** p < 0.01, * p < 0.05). DF- days to flowering; DPB-days to pod beginning; DSF- days to seed formation; DMI- days to maturity initiation. 18 and 19 represent the years of 2018 and 2019, DT : Dangtu; JP : Jiangpu; 2019YC6 and 2019YC7 means the planting date of June and July in Yancheng city, respectively. [file Data_Sheet_3.docx]

**
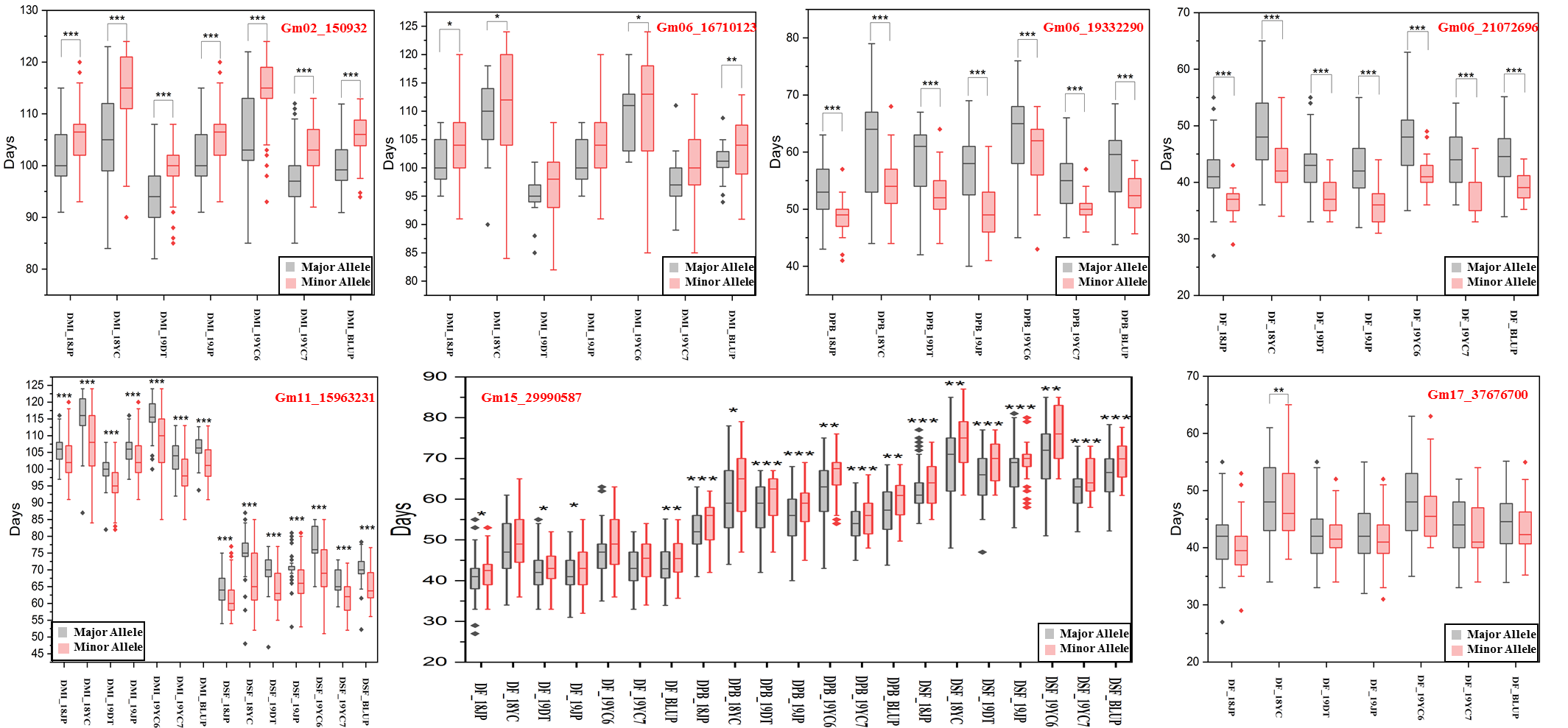
**

**Supplementary Figure 3. Phenotypic variation between cultivars carrying different alleles of the QTNs significantly associated with four growth stage traits in various environments.** The box plot shows the significant difference of days to flowering, pod beginning, seed formation and maturity initiation of the cultivars carrying two alleles of the QTNs. The significant QTNs were *Gm02_150932*, *Gm06_16710123*, *Gm06_19332290*, *Gm06_21072696*, *Gm11_15963231*, *Gm15_29990587* and *Gm17_37676700*. The major allele of significant loci was marked by grey, and the minor allele was marked by pink. Significant differences tested by the t-test are also given (****p* < 0.001, ***p* < 0.01, **p* < 0.05). DF- days to flowering; DPB-days to pod beginning; DSF- days to seed formation; DMI- days to maturity initiation. 18 and 19 represent the years of 2018 and 2019, DT : Dangtu; JP : Jiangpu; 2019YC6 and 2019YC7 means the planting date of June and July in Yancheng city, respectively.
